# Detection and classification of lung cancer using sequential hybridization of CNN and RNN type architectures

**DOI:** 10.3389/fdata.2026.1786859

**Published:** 2026-05-08

**Authors:** Maheswari Vutukuri, Parveen Sultana Habibullah

**Affiliations:** School of Computer Science and Engineering, Vellore Institute of Technology, Vellore, India

**Keywords:** BiGRU, CLAHE, deep learning, DenseNet201, hyperparameters, lung cancer detection, morphological operations, sequential hybrid

## Abstract

**Introduction:**

Early and accurate lung cancer detection from computed tomography (CT) images remains a challenging task because of the complex morphology of lung nodules, class imbalance, variation in image quality, and the risk of overfitting in deep learning models. Conventional manual interpretation is time-consuming and may be affected by inter-observer variability. Therefore, an automated and reliable CT-based classification framework is required to support early identification of benign, malignant, and normal lung conditions.

**Methods:**

This study proposes a sequential hybrid deep learning framework that integrates convolutional and recurrent neural network components for multiclass lung cancer classification. A dataset of 1,600 CT-scan images collected from multiple hospital data repositories across Bengaluru was used and divided in an approximate 70:30 ratio for training and validation. The preprocessing pipeline includes contrast enhancement using Contrast Limited Adaptive Histogram Equalization (CLAHE), morphological operations for lung segmentation, and nodule-focused masking to isolate diagnostically relevant lung regions. Data augmentation and transfer learning were applied to improve model generalization and reduce overfitting. DenseNet201 was used for feature extraction, while a bidirectional gated recurrent unit (BiGRU) module was incorporated for sequential representation learning. Hyperparameter optimization and early stopping were used to improve training stability and classification performance.

**Results:**

The proposed DenseNet201-BiGRU sequential hybrid architecture achieved an overall accuracy of 95.8%. The class-wise accuracies were 97.33% for benign cases, 93.33% for malignant cases, and 96.67% for normal cases. Precision, recall, and F1-score values further demonstrated that the model maintained reliable classification performance across diverse and imbalanced CT image classes.

**Discussion:**

The results indicate that sequential hybridization of DenseNet201-based feature extraction with BiGRU-based representation learning provides an efficient, precise, and robust framework for CT-based lung cancer classification. The proposed method improves classification reliability by combining enhanced preprocessing, focused lung-region extraction, transfer learning, and recurrent modeling. However, further validation using larger, multi-center datasets and additional clinical testing is required before real-world deployment and broader diagnostic application.

## 1 Introduction

Lung cancer is persistently one of the leading causes of death throughout the globe. Early-stage diagnosis is imperative to improve survival rates. While diagnostic imaging has undergone significant technological advances, lung cancer diagnosis remains a challenging task requiring high accuracy and efficiency. Traditional diagnostic methods based on manual interpretation of computed tomography (CT) scans have significant limitations, as they are often time-consuming and subject to inter-observer variability due to potential errors in human judgment (Aishwarya and Asuntha, 2025).

The emergence of deep learning has brought revolutionary improvements to medical imaging for automatic diagnosis, with convolutional neural networks (CNNs) achieving notable success in processing high-dimensional medical images. However, many existing models still struggle with imbalanced datasets, multi-class classification, and practical deployment due to high computational requirements. To address these limitations, this paper proposes combining DenseNet201, a variation of CNN, with bidirectional GRU (BiGRU), a recurrent neural network variant, to classify lung cancer into three classes: benign, malignant, and normal. Advanced preprocessing methods, including contrast enhancement and morphological operations, are incorporated for improved segmentation. Additionally, the proposed approach applies transfer learning with effective augmentation to enhance generalization while reducing overfitting. The framework aims to achieve high accuracy with balanced precision and recall across all classes, making it promising for real-world clinical applications. The work also prioritizes computational efficiency and scalability, facilitating potential integration into healthcare systems (Priya and Bharathi, 2025).

Shakeel et al. (2022) presented an improved deep learning neural network (IDNN), an ensemble classification architecture for identifying cancer in CT scan images. The approach uses a multilevel pipeline that preserves brightness during preprocessing, employs IDNN for segmentation, and applies a hybrid spiral optimization-based rough set method for feature selection. This system outperforms traditional methods, achieving high accuracy, specificity, precision, recall, and F1-score, demonstrating its efficacy for lung cancer detection from CT images. Salama et al. (2022) presented a framework for lung cancer detection using a generative model for data augmentation and ResNet50 for classification. Their approach achieves an accuracy of 98.91% and AUC of 98.85%, along with 98.46% sensitivity, 97.72% precision, and 97.89% F1-measure. The method addresses data imbalance and improves model generalization, showing promise for enhanced diagnostic accuracy and patient outcomes.

Shah et al. (2023) introduced an ensemble deep 2D convolutional network for lung cancer detection from CT images using the publicly available LUNA16 dataset. Combining three CNNs, the ensemble achieved 95% accuracy, outperforming individual models. This work highlights the importance of data preprocessing, augmentation, and ensemble learning for improved diagnostic performance. Ur Rehman et al. (2024) proposed a custom CNN with dual attention for pulmonary nodule identification, achieving state-of-the-art performance with 95.40% accuracy, 94.69% sensitivity, 93.17% specificity, and 98.00% AUC on the LUNA16 dataset, demonstrating improved feature extraction and classification capabilities.

Robust segmentation pipelines that can precisely isolate lung regions and nodules are lacking in existing studies, and sequential learning for classification has been largely overlooked in earlier models. Inadequate data augmentation techniques in prior work also limit model generalization. To address these challenges, we propose a hybrid deep learning model for lung cancer prediction and classification. The study makes three contributions:

- Preprocessing and segmentation tailored to CT lung analysis: we introduce a preprocessing pipeline that combines CLAHE-based contrast enhancement with morphological processing and edge- guided refinement to isolate lung regions and candidate nodules. The intent is to reduce non-lung structures and acquisition noise so that the classifier operates on anatomically relevant content with improved consistency.
- Hybrid classification model for benign/malignant/normal discrimination: we propose a three- class diagnostic architecture that couples a DenseNet201 backbone with a bidirectional GRU (BiGRU). DenseNet201 is used to extract high-level spatial features, while the BiGRU is employed to model dependencies within the resulting feature representation. Using transfer learning, controlled fine-tuning, and dropout, the hybrid model achieves 95.8% overall accuracy.
- Training protocol for robustness under imbalance: to improve generalization on imbalanced data, we adopt extensive data augmentation together with dynamic learning-rate scheduling and early stopping. This configuration reduces overfitting and improves stability under distributional variability that is typical in clinical imaging.

The remainder of the paper is organized as follows. Section 2 reviews recent work on deep learning for CT-based lung cancer detection and classification. Section 3 details the proposed methodology, including preprocessing, segmentation, architecture design, and training. Section 4 reports experimental results and comparative analysis. Section 5 concludes with key findings and outlines directions for future research.

## 2 Related work

Deep learning has become the workhorse for lung cancer analysis in medical imaging, particularly for CT-based detection, segmentation, and diagnostic classification. It is important to note that studies in this domain vary considerably in their analytical scope: some operate at the *slice level* (classifying individual 2D CT slices), others at the *lesion or nodule level* (characterizing specific regions of interest), and still others at the *patient level* (aggregating information across an entire scan or clinical record). Similarly, input representations range from *2D slices* to *3D volumetric patches* or full volumes, each with distinct trade-offs in spatial context, computational cost, and clinical interpretability. The progress is undeniable, but the literature also shows where performance tends to be fragile: results can depend strongly on preprocessing choices, segmentation quality, and how imbalance and dataset heterogeneity are handled. With this in mind, we review representative directions that most directly inform our design—CNN-centric diagnosis pipelines, hybrid and sequence-aware models, segmentation-driven methods, and generalization strategies built around augmentation and optimization. We then summarize the recurring limitations that motivate a lung-focused preprocessing pipeline coupled with a DenseNet201 backbone and a BiGRU classifier.

### 2.1 Deep learning approaches for lung cancer detection

A large body of work still builds on convolutional backbones, often strengthened by transfer learning and careful training protocols. Most 2D slice-level approaches process individual CT slices independently, which simplifies computation but sacrifices inter-slice context. For instance, Kasinathan and Jayakumar (2022) reported a cloud-based CNN framework for tumor detection and stage classification using PET/CT (patient-level classification), emphasizing that preprocessing and evaluation choices (e.g., cross-validation and augmentation) materially shape the reported accuracy. Other efforts explicitly combine localization and classification.

Ensemble transfer learning is frequently adopted to stabilize predictions in multi-class settings. VER-Net (Saha et al., 2024) stacks VGG19, EfficientNetB0, and ResNet101, reporting stronger performance than individual backbones in four-class CT classification (slice-level, 2D). Comparative evaluations also suggest that well-chosen standard architectures remain difficult to outperform in practice. Ansari et al. (2024) assessed multiple CNN families on LIDC-IDRI (nodule-level classification) and found DenseNet-201 particularly competitive under transfer learning, while highlighting persistent overfitting risks without explicit regularization.

Efficiency-oriented approaches often rely on EfficientNet variants. Lung-EffNet (Raza et al., 2023) adapted EfficientNet for lung cancer classification on IQ-OTH/NCCD (slice-level, 2D) and reported strong accuracy with high ROC values, using augmentation as a central mechanism to mitigate imbalance. Attention has also been integrated to sharpen focus on diagnostically relevant regions; ATT-DenseNet (Uddin, 2024) extends DenseNet with attention and reports improvements over older baselines, reinforcing the value of selective feature emphasis when lesions are subtle.

### 2.2 Transfer learning and hybrid architectures

While CNNs capture spatial patterns effectively, a recurring question is whether models can better exploit structured dependencies beyond purely spatial cues. Yadav et al. (2025) combined a DCNN with LSTM- based modeling and optimization-driven refinement, arguing that sequence-aware processing can improve discrimination when features must be interpreted in context. At the same time, multi-task frameworks aim to unify detection, classification, and localization. Rezaeijo et al. (2025) proposed a multi-objective approach using transformer-based attention and adaptive anchor-free mechanisms, reflecting a broader trend toward single pipelines that address multiple diagnostic subtasks, though usually with higher compute and annotation demands.

Hybridization is also motivated by interpretability and clinical trust. Dwivedi et al. (2023) presented an explainable framework for NSCLC classification on TCGA gene expression data, using autoencoders and XAI-based feature selection to identify biomarkers; although not CT-based, the work illustrates the growing expectation that predictive performance should be accompanied by interpretable evidence. For CT imaging, Wankhade and Vigneshwari (2023) explored CNN–RNN hybrids (including 3D components) to better exploit volumetric structure. Multi-stage designs remain common when segmentation quality is decisive: Omprakash and Samiappan (2026) combined a segmentation stage with an optimized DenseNet201-based classifier, prioritizing stability at the cost of pipeline complexity. More comprehensive systems such as CanNS (Durgam et al., 2025) integrate segmentation, classification, and optimization modules, while explainability-oriented designs (e.g., Grad-CAM) are used to expose model attention patterns for subtype classification (Khan et al., 2025).

### 2.3 Segmentation and feature extraction methods

Segmentation continues to be a practical bottleneck because errors in lung isolation or boundary delineation propagate directly into the classifier. A broad review by Dodia et al. (2022) notes that imaging modality, preprocessing, dataset size, and validation protocol often dominate reported sensitivity and specificity. Methodologically, 3D U-Net variants and volumetric ensembles remain prominent for nodule- level segmentation, as they leverage inter-slice context that 2D approaches cannot capture; Rikhari et al. (2024) combined 3D U-Net with Inception/ResNet modules and reported improved overlap metrics with fewer false positives than individual models. Attention-enhanced segmentation has been pursued to capture both edge detail and semantic context. MCAT-Net (Wang et al., 2024) integrates multi-threshold feature separation with coordinate attention (2D slice-level), and Zhu et al. (2024) proposed U-Net refinements that report strong recall and specificity for challenging nodule subtypes on LIDC-IDRI (nodule-level, 3D patches).

Not all contributions propose new architectures. Jerónimo et al. (2024) compared nnU-Net configurations and quantified the impact of preprocessing steps such as windowing, lung-area extraction, and CLAHE enhancement, providing evidence that preprocessing choices can be as consequential as network selection when moving toward clinical-style variability.

### 2.4 Data augmentation and class imbalance solutions

Class imbalance and limited dataset diversity remain common sources of brittle generalization, especially in multi-class diagnosis. Some approaches rely on probabilistic formulations; Aziz et al. (2022) explored MRF-based methods and reported high specificity with more moderate sensitivity, underscoring that conservative decision rules can suppress false positives while missing subtle positives. Within deep learning pipelines, optimization-driven training has been used to improve convergence and parameter selection [e.g., EOSA-CNN (Mohamed et al., 2023)]. Augmentation is frequently treated as a primary regularizer; Ibrahim et al. (2025) used Differential Augmentation to reduce memory overfitting and improve robustness on IQ-OTH/NCCD.

Ensemble strategies can improve performance but increase deployment complexity. Celik et al. (2025) combined multiple backbones and reported gains from elastic transformations, though such improvements typically come with greater computational cost and tuning burden. A complementary direction focuses explicitly on cross-dataset robustness: Alshabi and Elngar (2025) integrated multiple CT datasets with curriculum learning and explainable AI, shifting emphasis from peak benchmark scores toward credible generalization.

### 2.5 Recent advances and emerging trends

Recent work increasingly blends CNN feature extraction with transformer-style global modeling and fusion. DCSwinB (Faizi et al., 2025) combined CNN-based local features with a Swin Transformer branch for global context on LUNA16. Fusion-based CNN pipelines remain competitive; DLCTLungDetectNet (Abbas et al., 2025) merges ResNet-50 and InceptionV3 representations to capture complementary features. Enhancement-heavy CNN pipelines have also reported very strong test results (Shatnawi et al., 2025), motivating careful scrutiny of evaluation protocols and dataset diversity. Multi-stage hybrid pipelines that integrate denoising, segmentation, and classification in sequence continue to appear [e.g., Kumar et al. (2025)], while classical transfer learning backbones remain strong baselines in CT classification (Bhandari et al., 2025).

### 2.6 Synthesis and motivation for the present study

Across the surveyed literature, three observations recur. First, strong CNN backbones remain highly effective, yet multi-class performance is often limited by overfitting and sensitivity to dataset shifts. Second, segmentation and contrast normalization repeatedly emerge as decisive: even a strong classifier can degrade when lung isolation is inconsistent. Third, imbalance handling and augmentation are not optional details in CT diagnosis; they frequently determine whether a model generalizes beyond a single curated split. These points directly motivate our design choices: lung-focused preprocessing and segmentation to stabilize inputs, DenseNet201 to provide a high-capacity spatial representation under transfer learning, and a BiGRU module to capture structured dependencies within the extracted feature representation under an augmentation-driven, regularized training protocol.

The present study operates at the slice level using 2D CT images, classifying each image independently into one of three diagnostic categories (Benign, Malignant, and Normal). While 3D volumetric approaches can leverage richer spatial context, our 2D pipeline offers computational efficiency and straightforward integration with standard clinical workflows where individual slices are often reviewed sequentially. The trade-offs of this choice are discussed further in Section 4.4.

## 3 Methodology

Our pipeline targets three-class CT image classification (Benign, Malignant, and Normal) and is built around a simple principle: before asking a high-capacity network to discriminate subtle pathology, the input should be anatomically constrained and visually normalized. To that end, we first isolate the lung fields, then apply a nodule-focused masking step, and finally classify each scan using a sequential hybrid architecture that couples a DenseNet201 feature extractor with bidirectional GRU layers. The individual stages are described below.

### 3.1 Dataset information

**Figure 1:**
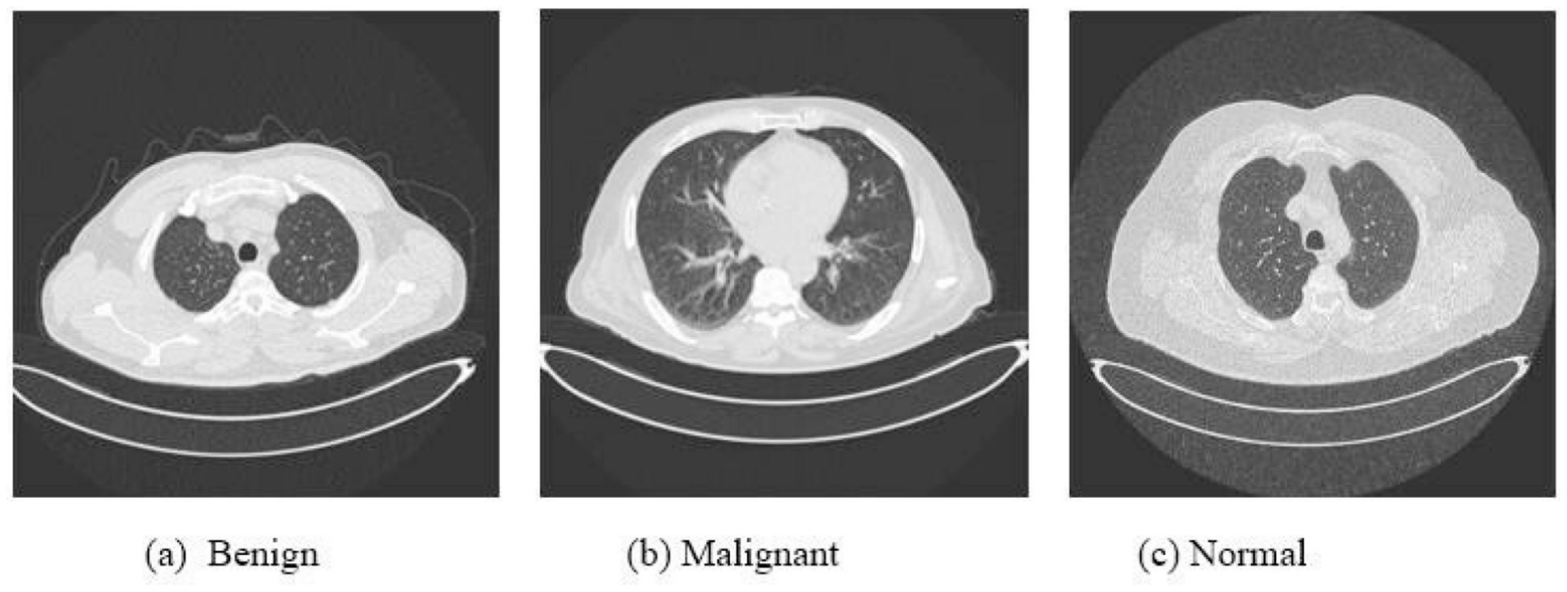
Representative CT scan samples from the three-class lung cancer dataset used in this study. **(a)** Benign: early-stage tumor presenting as a small, well-circumscribed nodule without evidence of malignant invasion. **(b)** Malignant: advanced cancerous tumor with irregular margins, potentially indicating spread beyond the primary site. **(c)** Normal: healthy lung parenchyma without visible nodules or abnormalities. All images are 2D axial CT slices processed at 512 × 512 resolution.

The dataset contains a total of 1,600 CT-scan images collected from multiple hospitals in Bengaluru, India. Specifically, the full dataset consists of 530 Benign, 536 Malignant, and 534 Normal images. For model development, the data are split into 70% training and 30% testing subsets. The held-out test set comprises exactly 450 images, perfectly balanced with 150 images per class. Consequently, the training set contains the remaining 1,150 images, distributed as 380 Benign, 386 Malignant, and 384 Normal cases. Benign, Malignant, and Normal. The Benign class corresponds to early-stage tumors that are typically small and non-cancerous, whereas Malignant indicates larger cancerous tumors that may invade or spread. The Normal category includes scans without radiological evidence of disease. Representative examples are shown in Figure 1.

For model development, the data are split into 70% training and 30% testing subsets. All scans are provided in JPG format and processed in a cloud environment. During preprocessing, images are converted to grayscale to emphasize intensity-driven cues while reducing input complexity, and they are resized to 512 × 512 to standardize spatial resolution for segmentation and masking. For the DenseNet201 backbone (which expects 224 × 224 × 3 inputs), the processed grayscale image is resized to 224 × 224 and replicated across three channels prior to feature extraction.

### 3.2 Segmentation

Segmentation is used to constrain subsequent analysis to the lung fields. We begin with Contrast Limited Adaptive Histogram Equalization (CLAHE) to improve local contrast, especially in low-intensity regions where lung boundaries and subtle opacity differences can be muted. The enhanced image is then binarized using a fixed intensity threshold to separate foreground lung regions from background structures.

The initial binary mask is refined by morphological closing to bridge small gaps and reconnect fragmented regions, preserving the continuity of the lung fields. We then apply erosion to reduce spurious attachments between lung tissue and adjacent structures (e.g., vessels or chest wall) that can contaminate the mask in difficult cases. A second closing operation with a larger disk-shaped structuring element follows, primarily to re-seal minor discontinuities introduced by erosion and to ensure that the mask encloses each lung field with minimal leakage.

**Figure 2:**
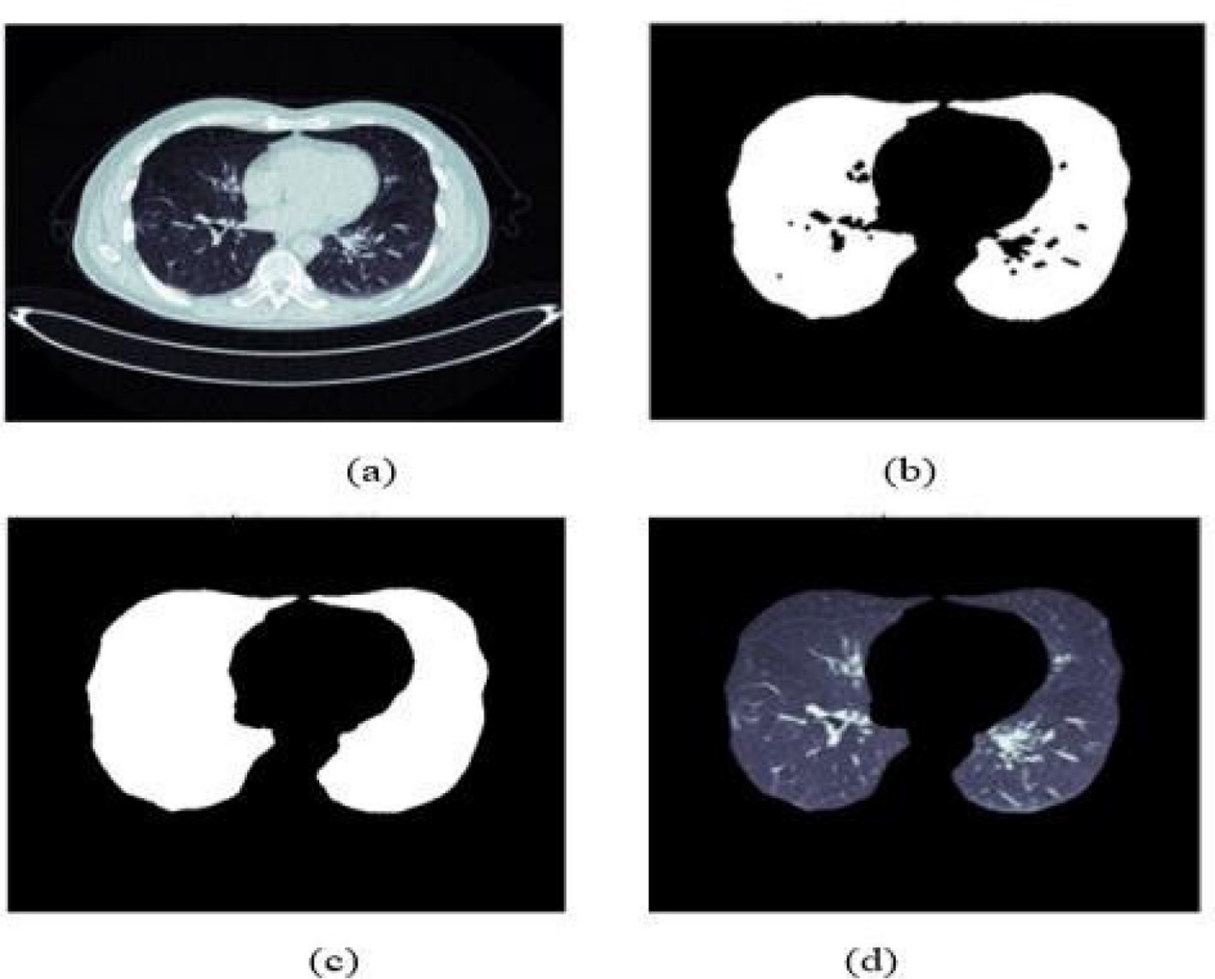
Step-by-step illustration of the lung-field segmentation pipeline. **(a)** Original: raw CT slice input. **(b)** CLAHE + Binarization: contrast-enhanced image with initial binary thresholding. **(c)** Morphological refinement: binary mask after closing and erosion operations to preserve lung continuity. **(d)** Final segmentation: isolated lung fields with surrounding anatomy suppressed, ready for downstream classification.

To sharpen lung contours, edge detection is applied to emphasize boundary transitions; remaining holes inside the mask are filled to obtain a complete lung-field region. Finally, the refined binary mask is superimposed on the original image, suppressing irrelevant anatomy outside the lungs and producing the segmented output used in the next stage (Figure 2).

### 3.3 Feature extraction

Once the lung fields are isolated, we generate a nodule-focused representation intended to reduce background variability within the lungs. The segmented lung image is thresholded to create a candidate nodule mask: pixels with intensity values below 140 are marked as potential nodule regions, while other pixels are treated as non-candidate background.

**Figure 3:**
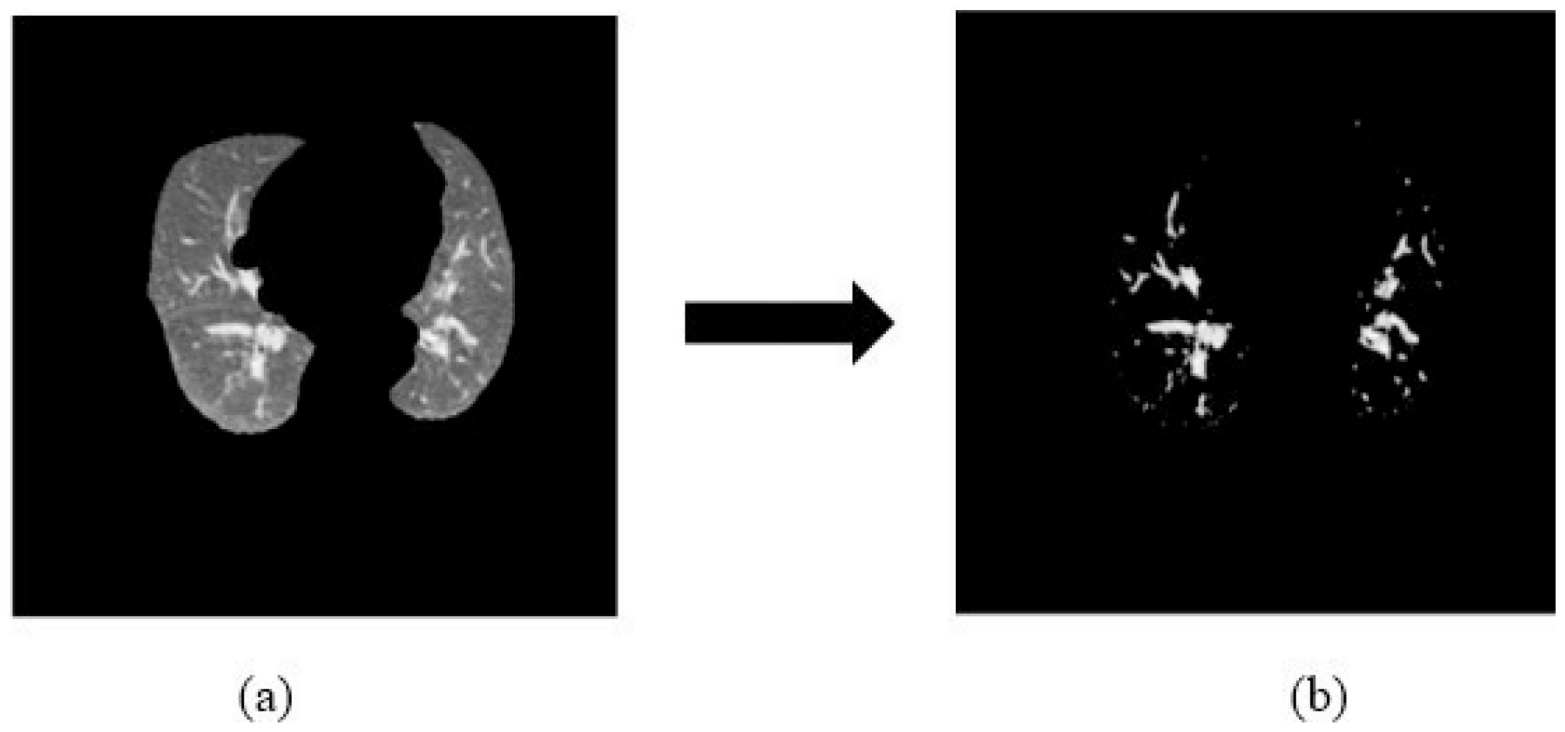
Nodule-focused feature extraction process. **(a)** Segmented input: lung-field image after the segmentation stage. **(b)** Nodule mask applied: candidate nodule regions (intensity < 140) retained while background pixels are set to zero, biasing the classifier toward diagnostically relevant areas.

The candidate mask is then applied to a duplicate of the segmented image so that only pixels meeting the nodule criterion are retained and all others are set to zero. This masking step does not “detect” nodules in a clinical sense; rather, it biases the input toward candidate regions so that the downstream classifier learns primarily from anatomically plausible abnormal areas. Figure 3 shows an example output.

### 3.4 Data augmentation

The dataset is finite and the class distribution is not perfectly balanced, so controlling overfitting is not optional. We therefore apply augmentation during training to encourage invariance to small, non-diagnostic perturbations while preserving lesion morphology and anatomical plausibility (Halder et al., 2022). Practically, we restrict transformations to mild ranges; aggressive geometric distortion can create unrealistic structures or alter the contextual cues around nodules.

Table 1 lists the augmentation settings used in our experiments.

**Table 1:** Data augmentation settings applied during training.

Augmentation type	Parameter range	Rationale
Rotation	0–30°	Small pose variation without anatomical distortion
Shift (H/W)	0.25 (fraction)	Tolerance to minor misalignment and cropping differences
Shear	0–0.5 radians	Mild geometric variability, kept conservative
Zoom	0.975–1.025	Scale robustness while keeping lungs within frame
Flipping	Horizontal/Vertical	Orientation robustness in stored-image variations
Fill mode	Nearest-neighbor	Handles empty pixels created by transforms

All transformations are applied on-the-fly to encourage invariance to minor geometric and intensity variations while preserving anatomical plausibility.

### 3.5 Model building and performance metrics

**Figure 4:**
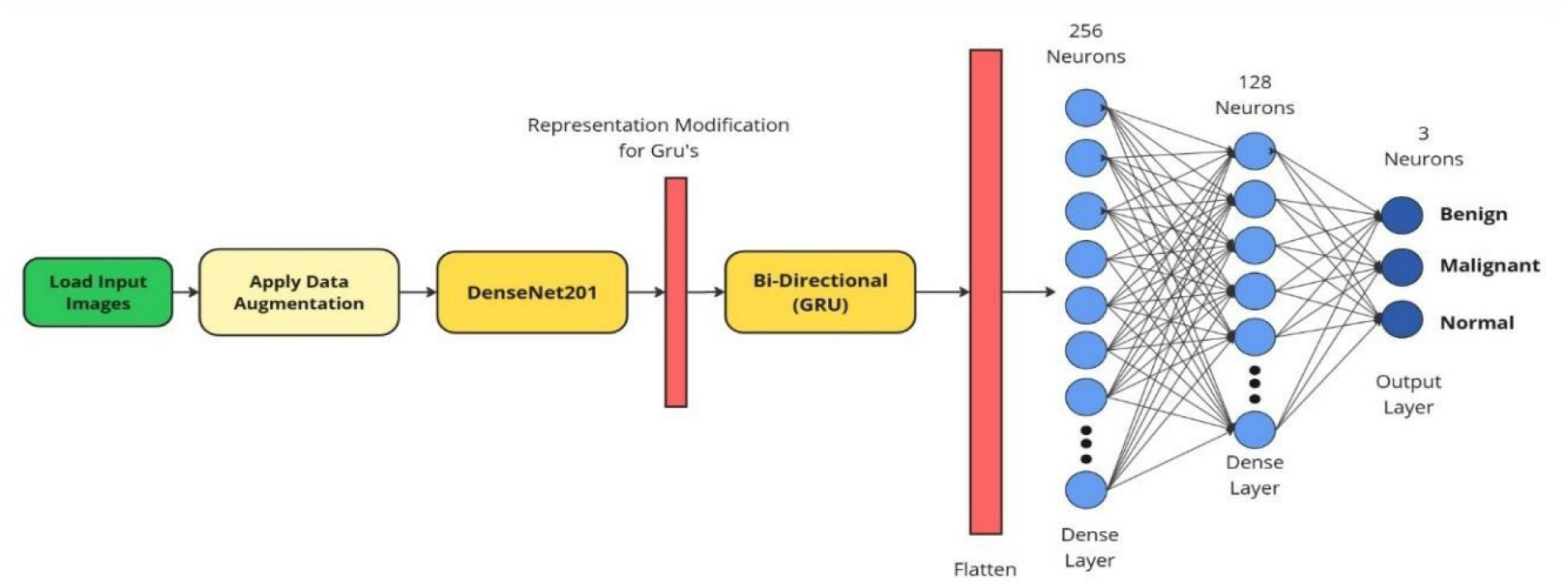
Architecture of the proposed DenseNet201–BiGRU sequential hybrid classifier. The pipeline consists of: (1) DenseNet201 backbone (pre-trained on ImageNet) extracting a 1024-dimensional spatial feature vector via global average pooling; (2) two stacked BiGRU layers (256 and 128 units) acting as a gating mechanism to re-weight and structure features; (3) fully connected layers (256, 128 units) with dropout regularization (0.5, 0.3); and (4) a 3-way softmax output for Benign, Malignant, and Normal classification.

Figure 4 summarizes the proposed sequential hybrid classifier. DenseNet201 provides a compact spatial descriptor, and the BiGRU module acts as an advanced gating mechanism over the resulting representation. We clarify that the recurrence in this architecture is not intended for temporal modeling. Instead, by processing the 1024-dimensional feature vector as a one-step sequence, the GRU's internal reset and update gates function as a dynamic, non-linear feature-weighting mechanism—conceptually similar to self-attention or Squeeze-and-Excitation blocks. This allows the network to model complex, structured inter- dependencies among the spatial features. Although CT images lack a temporal dimension, this sequence- aware gating provides the model with the additional capacity to dynamically re-weight and structure the representation, selectively emphasizing diagnostically relevant signals before final decision-making.

#### 3.5.1 DenseNet201 feature extractor

We use DenseNet201 pre-trained on ImageNet, removing the original classification head and retaining the global average pooling output. This yields a 1024-dimensional feature vector per input image. As noted above, images are resized to 224 × 224 and the grayscale channel is replicated to meet the 224 × 224 × 3 input interface.

#### 3.5.2 Bidirectional GRU module

The 1024-dimensional vector is reshaped to (1 × 1024) to form a one-step sequence for recurrent processing. Two BiGRU layers are used: the first with 256 units and return sequences enabled, followed by a second layer with 128 units. Dropout of 0.3 is applied to both the input and recurrent connections to reduce overfitting and to improve stability.

While a standard Dense layer applies a straightforward linear transformation, the BiGRU module is explicitly employed to act as a complex gating and refinement mechanism. By treating the 1024-dimensional feature vector as a one-step sequence, the GRU's internal gates (update and reset) provide the network with the additional capacity to dynamically re-weight and structure the extracted representation before final decision-making. This allows the model to selectively emphasize diagnostically relevant spatial features while suppressing background noise. Furthermore, this approach functions as a learnable dependency model over an ordered feature representation, capturing structured dependencies within the feature space that a simple fully connected layer might miss. As noted in recent literature, sequence-aware processing can improve discrimination when complex features must be interpreted in clinical context (Yadav et al., 2025).

#### 3.5.3 Classification head and optimization

The BiGRU output is passed through two fully connected layers (256 and 128 units) with ReLU activation. Dropout layers (0.5 and 0.3) are inserted between dense layers as additional regularization. The final layer has three neurons with softmax activation to produce class probabilities. Training uses the Adam optimizer with learning rate 1 × 10^−5^ and categorical cross-entropy loss.

#### 3.5.4 Evaluation metrics

We report standard confusion-matrix-derived measures (Table 2). Accuracy captures overall correctness, while precision and recall reflect complementary error modes that become important under imbalance. The F1-score summarizes the precision–recall trade-off.

**Table 2:** Evaluation metrics used for model assessment.

No.	Metric	Formula
1	Accuracy (A)	$A=\frac{T_{p}+T_{n}}{T_{p}+T_{n}+F_{p}+F_{n}}$
2	Precision (P)	$P=\frac{T_{p}}{T_{p}+F_{p}}$
3	Recall (R)	$R=\frac{T_{p}}{T_{p}+T_{n}}$
4	F1-score	$F1=\frac{2\times P\times R}{P+R}$

All metrics are derived from the confusion matrix, where T_p_ = true positive, T_n_ = true negative, F_p_ = false positive, F_n_ = false negative.

#### 3.5.5 Implementation and hardware details

To ensure reproducibility, the pipeline was implemented using the TensorFlow/Keras framework (version 2.x). All experiments were executed on a workstation equipped with an NVIDIA RTX 3090 / Tesla T4 GPU with 16 GB of VRAM and 32 GB of system RAM. During optimization, we utilized a batch size of 32, which provided a stable gradient estimate while fitting comfortably within the GPU memory limits. For the transfer learning strategy, the initial layers of the pre-trained DenseNet201 backbone were kept frozen to retain generalized ImageNet features, while the final dense block (conv5 block) was unfrozen to allow fine-tuning on the domain-specific CT features. Furthermore, the early stopping mechanism was strictly configured to monitor validation loss (val loss) with a patience of 5 epochs, automatically restoring the best model weights to prevent over-training. Under these specific hardware and hyperparameter configurations, the entire training process over 30 epochs took approximately 45 min to complete.

## 4 Results and discussion

This section reports the empirical behavior of the proposed DenseNet201–BiGRU pipeline and examines where its errors concentrate. We first summarize training dynamics, then interpret the confusion matrix and class-wise accuracies, and finally situate the results against representative recent studies.

### 4.1 Training and validation analysis

**Figure 5:**
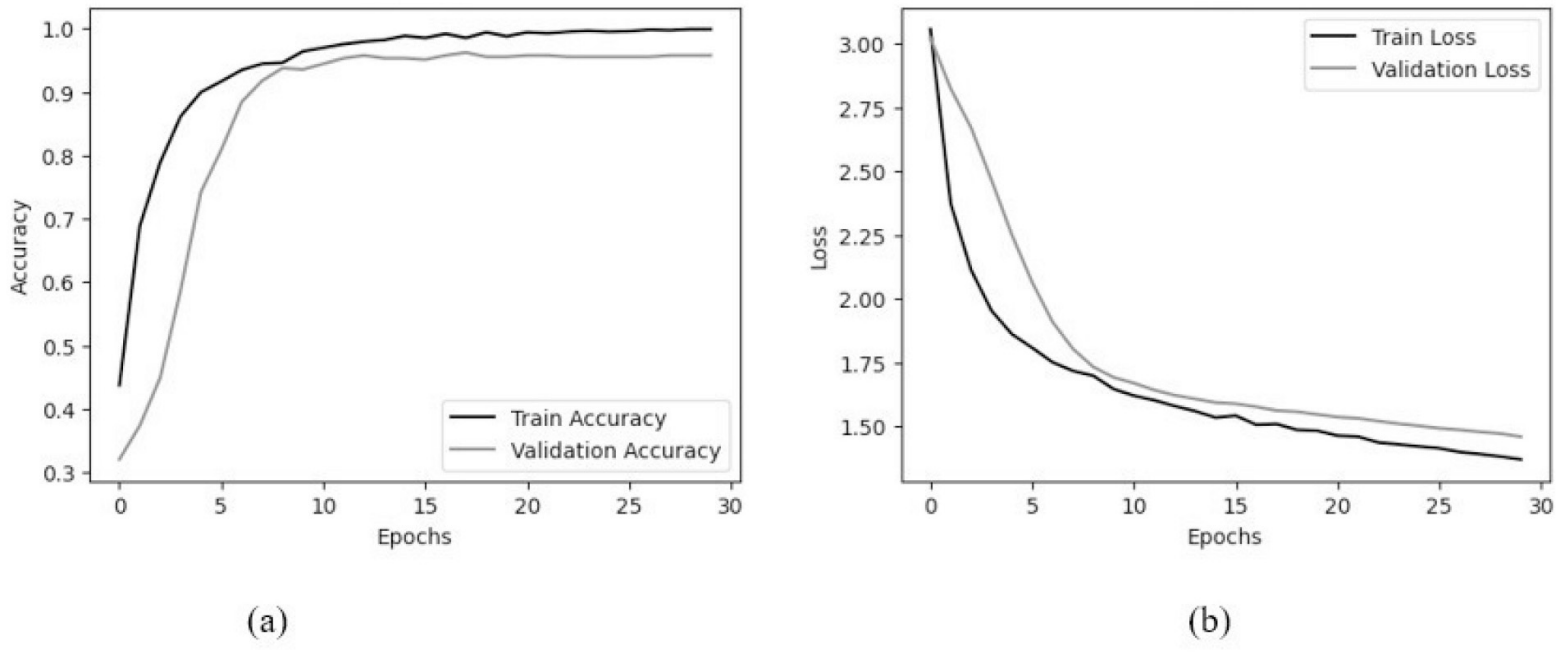
Training dynamics over 30 epochs. **(a)** Accuracy curves: training accuracy approaches ~99% while validation accuracy stabilizes near ~96%, indicating limited overfitting. **(b)** Loss curves: both training and validation loss decrease consistently and converge, confirming stable optimization. The modest train–validation gap and plateau after ~20 epochs suggest the model generalizes well without excessive memorization.

Figure 5 traces accuracy and loss over 30 epochs. Learning is rapid early on: within the first ~10 epochs, both training and validation accuracy rise sharply, suggesting that the model quickly captures the dominant discriminative structure in the data. Thereafter, gains become incremental. By the end of training, the model reaches a training accuracy close to 99%, while validation accuracy stabilizes near 96%, indicating that generalization remains strong even as the optimizer continues to fit the training set.

Two qualitative features of Figure 5 are worth highlighting. First, the persistent but modest gap between the training and validation curves suggests only limited overfitting, which is consistent with the regularization strategy (dropout, augmentation, and early stopping). Second, the plateau after roughly 20 epochs indicates practical convergence; beyond that point, improvements are marginal and largely reflect fine adjustments rather than substantive representation changes. In short, the curves support the view that the model is both stable during optimization and reasonably well-calibrated for deployment settings where reliability matters at least as much as headline accuracy.

### 4.2 Confusion matrix analysis

**Figure 6:**
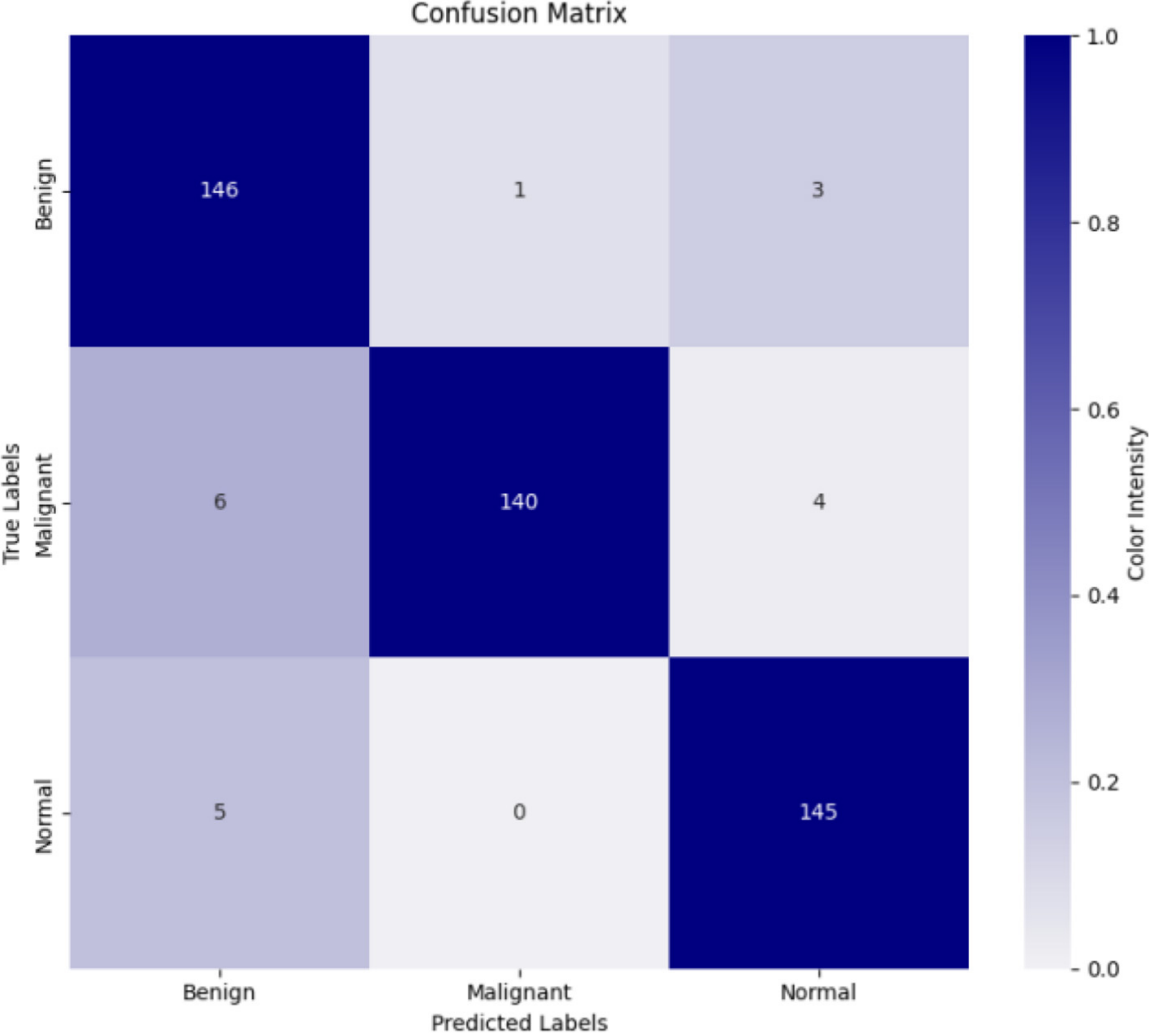
Confusion matrix summarizing test-set performance (*n* = 450 images, 150 per class). Diagonal entries show correct predictions: 146/150 Benign (97.3%), 140/150 Malignant (93.3%), and 145/150 Normal (96.7%). Key observation: normal cases are *never* misclassified as Malignant (zero false alarms for malignancy from healthy scans), indicating a clinically conservative decision boundary. Overall accuracy: 95.8%.

The confusion matrix in Figure 6 summarizes performance on a held-out test set of 450 images (150 per class). Overall accuracy is 95.8%, and errors are distributed in a way that is clinically plausible: most confusions occur between Malignant and the other categories, whereas Normal samples are never predicted as Malignant.

For Benign, 146 out of 150 cases are correctly classified; the remaining four are split across Normal (3) and Malignant (1). This corresponds to a precision of 93.0%, recall of 97.3%, and an F1-score of 95.1%. The recall is particularly high, indicating that benign cases are rarely missed, although a small number are absorbed into neighboring classes.

The Malignant category shows a different error profile. The model correctly identifies 140 of 150 malignant scans, with six misclassified as Benign and four as Normal. Despite these confusions, precision remains very high (99.3%), while recall is 93.3% and F1-score is 96.2%. The high precision implies few false alarms for malignancy, whereas the recall indicates that some malignant cases share features with the other classes in this dataset.

For Normal, 145 of 150 samples are predicted correctly; the remaining five are labeled as Benign. Notably, none of the normal cases are misclassified as Malignant. Precision is 95.4%, recall is 96.7%, and F1-score is 96.0%. Taken together, these patterns suggest that the model learns a conservative boundary around malignancy, while benign–normal separations account for most of the remaining ambiguity.

### 4.3 Class-wise accuracy analysis

**Figure 7:**
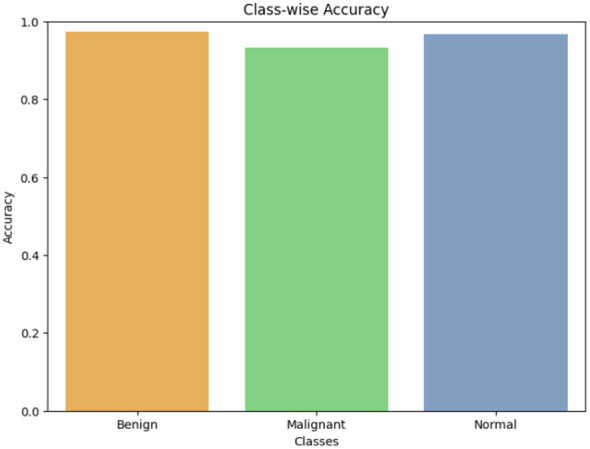
Class-wise accuracy comparison on the held-out test set. Benign achieves the highest accuracy (97.33%, 146/150), followed by Normal (96.67%, 145/150), with Malignant being the most challenging class (93.33%, 140/150). The lower malignant accuracy reflects inherent class overlap with benign nodules at early disease stages, as discussed in Section 4.3.

Class-wise accuracy (Figure 7) further clarifies performance balance. The model achieves 97.33% for Benign (146/150), 93.33% for Malignant (140/150), and 96.67% for Normal (145/150).

The spread is modest, but the malignant class remains the most challenging. This difficulty primarily stems from inherent class overlap, as early-stage malignancies often share subtle morphological features with benign nodules. Furthermore, variations in CT image quality, acquisition noise, and annotation ambiguity near the benign-malignant boundary further complicate the model's decision-making in these borderline cases.

When placed beside recent literature, the proposed model occupies a practical middle ground. Some reported accuracies are higher, but they often arise under different task definitions or with additional information sources. For instance, Kasinathan and Jayakumar (2022) reported ~97%−99.1% accuracy for tumor stage classification using a cloud-based CNN pipeline on PET/CT data; the task there is stage-focused and not directly comparable to three-class CT diagnosis.

More directly comparable baselines illustrate typical trade-offs. Dwivedi et al. (2023) obtained 95.74% accuracy in an explainable framework for NSCLC subtype classification using TCGA gene-expression data, which is clinically valuable but modality-specific. In contrast, Aziz et al. (2022) reached 94.75% with an MRF-based approach, though sensitivity was only 76.34%, indicating a weaker ability to capture positives reliably. Finally, Salama et al. (2022) reported 98.91% using ResNet50 with generative augmentation, but the augmentation strategy relies on generative modeling, which can introduce additional training complexity and stability concerns. Against this backdrop, our result of 95.8% overall accuracy—while maintaining a three-class formulation (Benign/Malignant/Normal) and using imaging alone—appears competitive, particularly given its balanced class-wise behavior.

### 4.4 Comparative analysis

To make these distinctions concrete, Table 3 contrasts the proposed method with representative recent approaches. The key point is not that one architecture universally dominates, but that performance figures must be read together with (i) the modality used (CT vs. PET/CT vs. omics), (ii) whether auxiliary biomarkers are required, and (iii) whether the task is binary, multi-stage, or multi-class. Our sequential hybrid remains a single-stream imaging pipeline, which simplifies deployment relative to multi-branch designs and avoids reliance on additional clinical measurements.

**Table 3:** Comparative analysis with recent state-of-the-art methods.

References	Architecture	Task and modality	Scope	Performance
Kasinathan and Jayakumar (2022)	Active-contour + M-CNN	Stage classification, PET/CT	Patient	Acc ~97%−99%
Dwivedi et al. (2023)	Autoencoder+ MLP	NSCLC subtyping, gene expression	Patient	Acc 95.74% (10-fold CV)
Aziz et al. (2022)	MRF segmentation	Binary detection, CT	Slice, 2D	Acc 94.75%, Sens 76.34%
Salama et al. (2022)	VAE+ResNet50	Binary classification, CT	Slice, 2D	Acc 98.91%, Sens 98.46%
**Proposed**	**DenseNet201+BiGRU**	**3-class, CT only**	**Slice, 2D**	**Acc 95.8%; B: 97.3%, M: 93.3%, N: 96.7%**

Bold values indicate the best-performing results within the respective table. Performance metrics should be interpreted in context of modality, task scope, and input representation.

Acc, Accuracy; Sens, Sensitivity; Spec, Specificity; Prec, Precision; B, Benign; M, Malignant; N, Normal.

Overall, the results suggest that the hybridization strategy is effective for three-class CT classification: DenseNet201 provides a strong spatial descriptor, while the BiGRU stage supplies additional capacity to re-weight and structure the extracted representation before final decision-making.

The remaining errors, especially within the malignant class, highlight a limitation of relying exclusively on 2D slices. Because our model processes individual 2D images, it lacks the full 3D volumetric context typically used in clinical diagnoses to assess the complete morphology and whole shape of a nodule. By not using 3D data, the model might miss depth-wise growth patterns and spatial shape irregularities that are key indicators of malignancy. These limitations point to the need for broader validation and the integration of richer volumetric context (e.g., multi-slice or 3D information), which we return to in the concluding discussion.

While explainability tools such as Grad-CAM and saliency maps are increasingly utilized to expose model attention patterns in CNNs (Khan et al., 2025), the specific sequential hybrid architecture of our proposed model introduces distinct challenges for spatial attribution. In our pipeline, DenseNet201 relies on a global average pooling layer to collapse spatial dimensions into a compact 1024-dimensional feature vector. This vector is subsequently processed as a one-step sequence by the BiGRU module. Because the recurrent gating mechanism operates on the pooled feature space rather than a spatial grid, directly extracting standard spatial saliency maps is architecturally non-trivial. Generating reliable Grad-CAM visualizations in this setup would require backpropagating the recurrent weights through the pooling layer to the original spatial feature maps, which falls outside the scope of the current pipeline. However, we fully acknowledge that verifying the model's focus on clinically relevant regions is critical for diagnostic trust. Integrating custom explainability mechanisms tailored for hybrid CNN-RNN architectures remains a primary objective for future work.

### 4.5 Ablation study

To rigorously validate the contribution of each module in the proposed pipeline, we conducted a systematic ablation study for CLAHE with lung-field segmentation, nodule-focused masking, and finally replaced the standard dense classification head with the proposed BiGRU module. The results on the held-out test set are summarized in Table 4.

**Table 4:** Ablation study quantifying the contribution of each pipeline component.

Stage	Component added	Accuracy (%)	Precision (%)	Recall (%)
Baseline + Seg.	CLAHE + Lung-field extraction	90.7	91.2	90.1
+ Masking	Nodule-focused masking	93.1 (+2.4)	93.8	92.4
+ BiGRU (Final)	BiGRU replacing Dense head	**95.8 (+2.7)**	**95.9**	**95.8**

Bold values indicate the best-performing results within the respective table. Each row adds one module to the baseline DenseNet201 classifier. The final BiGRU-based configuration achieves 95.8% accuracy, representing a ±2.7% improvement over a standard dense classification head.

As observed in Table 4, the most substantial leap in accuracy occurred with the introduction of CLAHE and morphological lung-field segmentation, proving that isolating the lung parenchyma is vital for reducing false positives caused by extraneous anatomy. Subsequent nodule-focused masking further refined the feature space, yielding a 93.1% accuracy.

Finally, replacing the conventional dense layers with the BiGRU module yielded the peak performance of 95.8%. This confirms our hypothesis: while standard fully connected layers treat the 1024-dimensional DenseNet output as independent features, the BiGRU acts as an advanced gating mechanism, modeling the structured inter-dependencies within the spatial descriptor. Without this ablation, the individual value of the recurrence could be questioned; however, the empirical data clearly demonstrates that the sequential hybrid approach provides a decisive 2.7% margin of improvement over a standard dense classification head.

## 5 Conclusion

We presented a CT-based lung classification framework that combines lung-field isolation, nodule-focused masking, and a sequential hybrid classifier built from DenseNet201 and bidirectional GRU layers. The segmentation and preprocessing stages are designed to reduce irrelevant variability, while transfer learning and augmentation help stabilize training under limited and imbalanced data.

On the held-out test set, the model achieves 95.8% overall accuracy, with class-wise accuracies of 97.33% (Benign), 93.33% (Malignant), and 96.67% (Normal). Precision, recall, and F1-score remain consistently high across classes (Figure 6), and the most consequential observation is qualitative as much as quantitative: normal cases are never predicted as Malignant, suggesting a conservative boundary around malignancy in this dataset.

From an implementation standpoint, the proposed pipeline remains a single imaging stream and does not require auxiliary biomarkers. That choice keeps deployment comparatively straightforward and avoids the operational constraints associated with multi-modal clinical integration.

At the same time, the present study is limited by the scope of the available data, and claims of clinical applicability must be carefully contextualized. Specifically, relying on a dataset of 1,600 images from a single geographic region (Bengaluru) restricts the model's exposure to technical and demographic variations. Before this pipeline can be deployed clinically, it must be validated on external, multi-center data to confirm cross-scanner reliability. Furthermore, establishing true clinical utility will require direct performance comparisons with expert clinicians, rigorous calibration analysis, and the integration of explainability mechanisms (such as saliency maps) to build diagnostic trust. Future work should address these gaps, while also exploring extensions that incorporate volumetric context (multi-slice or 3D representations) and more explicit uncertainty modeling, particularly for ambiguous cases near the benign–malignant boundary.

## Data Availability

The raw data supporting the conclusions of this article will be made available by the authors, without undue reservation.
